# Baicalin Protects Mouse Testis From Injury Induced by PM_2.5_ Associated With the DKK1‐Wnt/β‐Catenin Signaling Pathway

**DOI:** 10.1002/jbt.71057

**Published:** 2026-08-03

**Authors:** Lili Deng, Juan Wang, Shuyin Li, Yaping Mao, Ye Sun, Qiaoqiao Yang, Zhenghui Xie, Chen Zhang, Shuo Gong, Chunling Xiao, Mingyue Ma

**Affiliations:** ^1^ Department of Key Laboratory of Environmental Pollution and Microecology Shenyang Medical College Shenyang Liaoning Province China; ^2^ Department of Nutrition and Food Hygiene, School of Public Heath Shenyang Medical College Shenyang Liaoning Province China; ^3^ Health Services Section Administrative Functional Section, People's Hospital of LinXia, Hui Autonomous Prefecture of LinXia LinXia Gansu Province China; ^4^ Department of Health Inspection, School of Public Health Shenyang Medical College Shenyang Liaoning Province China; ^5^ Department of Toxicology, School of Public Heath Shenyang Medical College Shenyang Liaoning Province China; ^6^ School of First Clinical Shenyang Medical college Shenyang Liaoning Province China; ^7^ School of Public Health Wenzhou Medical University Wenzhou Zhejiang Province China

**Keywords:** baicalin, DKK1‐Wnt/β‐catenin pathway, PM_2.5_, reproductive damage

## Abstract

Fine particulate matter (PM_2.5_) has a damaging effect on the male reproductive system. Baicalin has excellent anti‐inflammatory and antioxidant pharmacological functions. The main purpose of this study was to investigate whether baicalin had a protective effect against PM_2.5_‐induced damage to the reproductive system of mice, and to further investigate the mechanism of the protective effect of baicalin. In our experiment, baicalin was administered by gavage 1 h before PM_2.5_ exposure, followed by exposure to PM_2.5_ with an oral‐nasal exposure system at 750 μg/m^3^ for 4 h/day, and this experiment was continued for 45 days until the model was established. The results indicated that PM_2.5_ exposure decreased testicular weight and organ coefficient, sperm count, and serum testosterone level, and down‐regulated the mRNA and protein expressions of StAR, CYP11A1, and CYP17A1, which related to testosterone synthesis. After baicalin treatment, our experimental results showed that the 50 and 100 mg/kg baicalin groups showed a gradual increase in sperm counts, testosterone levels, and a significant restoration of genes and protein related to testosterone synthesis and the DKK1‐Wnt/β‐catenin signaling pathway. In conclusion, baicalin might play a protective role in promoting testicular tissue repair, and this may be associated with the regulation of the DKK1 ‐ Wnt/β‐catenin signaling pathway.

## Summary

- Baicalin can improve the pathological damage of the reproductive system in mice by PM_2.5_ exposure.
- Baicalin can increase the expression of testosterone synthesis genes and proteins StAR, CYP11A1 and CYP17A1 in mice exposed to PM_2.5_, thereby promoting testosterone synthesis, improving the atrophy of testicular spermatogenic tubules, increasing the sperm count of mice, and enhancing sperm motility.
- The improvement mechanism of baicalin may be associated with the Wnt/β‐catenin signaling pathway.

## Graphical abstract

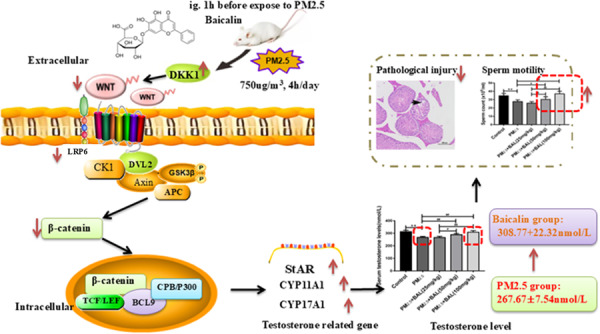

## 1 Introduction

Fine particulate matter (PM_2.5_) is the most important air pollutant. The main chemical components of PM_2.5_ are salts (sulfate and nitrate) and carbon [1]. PM_2.5_ is also easy to adsorb a variety of toxic substances (microplastics, polycyclic aromatic hydrocarbons, bacteria, viruses, etc.) and heavy metals (lead, cadmium, chromium, mercury, etc.) which can induce serious adverse health effects [2, 3]. Epidemiological studies [4, 5] have shown that sulfate concentration is closely related to mortality. Moreover, carbon components such as elementary carbon (EC) and organic carbon (OC) are prone to acute and chronic inflammation during their metabolism, leading to many diseases, including cardiovascular disease [5], asthma [6], lung cancer [6], Alzheimer's disease [7], diabetes mellitus [3], cognitive impairment [7] and low birth weight [8], reproductive diseases [9], and so on. Studies have shown that PM_2.5_ can cross the blood‐testosterone barrier and accumulate, disrupting hormone levels and ultimately affecting fertility, which mainly manifests as abnormal sperm motility and infertility [10, 11]. Henry et al. found that PM_2.5_ exposure is more damaging to early sperm production [12]. Qiu et al. analyzed the influence of PM_2.5_ exposure on the hypothalamic‐pituitary‐gonadal axis (HPG), confirming that PM_2.5_ can disrupt testosterone secretion and damage spermatogenesis by damaging HPG [13]. Therefore, it is important to explore measure to the protection of reproductive system injury, and the specific protective mechanism needs to be further explored. Wnt/β‐catenin is a signal pathway that plays a key role in development, injury, and repair [14, 15, 16]. In the classical Wnt pathway, Wnt ligands bind to Frizzled (FZD) receptors and LRP coreceptors and act by regulating kinases and transcription factors such as DVL, LRP5/6, GSK3b, APC/AXIN/CK1, β‐catenin, TCF/LEF [14]. Dickkopf‐associated protein‐1 (DKK1) is one of the inhibitors of the Wnt pathway. As part of the negative feedback loop, DKK1 antagonizes the Wnt pathway by inhibiting the interaction between the Wnt pathway and the membrane receptor LRP5/6, a typical inhibitor of the Wnt/β‐catenin signaling pathway [16]. Baicalin is a major flavonoid extracted from the dry root of *Scutellariabaicalensis*, which has a variety of pharmacological activities, such as antipyretic, sedative, anti‐inflammatory, and antioxidant [17, 18, 19, 20]. Zhang et al found baicalin promotes embryo adhesion and implantation by upregulating fucosyltransferaseIV (FUT4) via Wnt/β‐catenin signaling pathway [21]. The current study established a PM_2.5_ exposure model in mice, which was used to investigate the protective role of baicalin on reproductive system damage. We measured the body weight and organ coefficient of mice, the change of sperm number, the pathological and electron microscopic changes of mice testis, the testosterone level of different groups of mice, and the expression of testosterone synthesis pathway gene, and finally evaluated the protective effect of baicalin on the reproductive system. Furthermore, we investigated the mechanism of baicalin by determining the genes and proteins related to DKK1, Wnt, LRP5/6 and β‐catenin, signaling pathway in mouse testis.

## 2 Materials and Methods

### 2.1 Drugs and Reagents

Baicalin (purity ≥ 98%, specification:20 mg/piece) was purchased from Chengdu Mansit Biological Co. Ltd. (Chengdu, China). All primers were purchased from Bioengineering Co. Ltd.(Shanghai, China). Trizol was purchased from Shanghai Sangong Technology Co. Ltd. (Shanghai, China). The cDNA reverse transcription kit and SYBR Green fluorescent quantitative PCR kit were purchased from TaKaRa Company (Tokyo, Japan). RIPA protein lysate and BCA protein concentration determination kit were purchased from Shanghai Biyuntian Co. Ltd.(Shanghai, China). PAGE gel rapid preparation kit (7.5%) was purchased from Shanghai Yase Biological Co. Ltd.(Shanghai, China). Antibodies against DKK1, Wnt4 LRP5/6, β‐catenin, StAR, CYP17A1, CYP11A1, and β‐actin were all purchased from ABclonal Trading Co. Ltd. (Shanghai, China), Proteintech Group Inc. (Wuhan, China), and Abcam plc (Cambridge, USA). Horseradish peroxidase HRP labeled affinity purified goat anti‐rabbit IgG (H + L) and HRP anti‐mouse IgG (H + L) were purchased from Protein Technology Company (Wuhan, China) and ABclonal Trading Co. Ltd. (Shanghai, China).

### 2.2 Collection and Preparation of PM_2.5_

The traffic artery of Huanghe North Street in Shenyang, Liaoning Province was selected as the sampling point, and the sample particulate matter was collected on a nitrocellulose filter membrane (General Electric Company, GE, USA) with a flow rate of about 1 × 10^3^L/min for three consecutive months during winter season. The preserved filter membrane was cut into 1 cm×1 cm square, soaked in a large beaker with deionized water for elution with an ultrasonic oscillator, and filtered with six layers of sterile gauze, making a PM_2.5_ suspension (Ma et al., 2015). The suspension was centrifuged at 4°C, 12,000 g for 30 min to collect the precipitated particles, and these were stored at −80°C after freeze‐drying [22].

### 2.3 Animal Grouping and Sample Collection

The 7‐week‐old male BALB/cJ mice used in this study were purchased from Beijing Vital River Laboratory Animal Technology Co. Ltd. (license: SCXK [Beijing] 2016‐0011) (Beijing, China). The mice were raised in the SPF Experimental Animal Center of Shenyang Medical College, the breeding conditions meet the relevant requirements of the Laboratory Animal Environment and Facilities (GB 14925‐2010). They were kept at 22°C–24°C, 50%–60%, and 12 h/12 h diurnal‐night light cycle and were free to eat and drink. All experimental designs were approved by the Ethics Committee of Shenyang Medical College (SYYXY2021061502). After 1 week of adaptive feeding, 30 mice were randomly divided into five groups (*n* = 6/group): control group, PM_2.5_ exposure group (750 μg/m^3^), PM_2.5_+baicalin low dose group (25 mg/kg), PM_2.5_+baicalin medium dose group (50 mg/kg), PM_2.5_+baicalin high dose group (100 mg/kg). The doses of baicalin were selected based on previous research [23, 24, 25]and results of pre‐experiment. After poisoning, testicular tissue and biological sample materials were collected for follow‐up experiments.

### 2.4 PM_2.5_ Exposure and Drug Treatment

**Figure 1:**
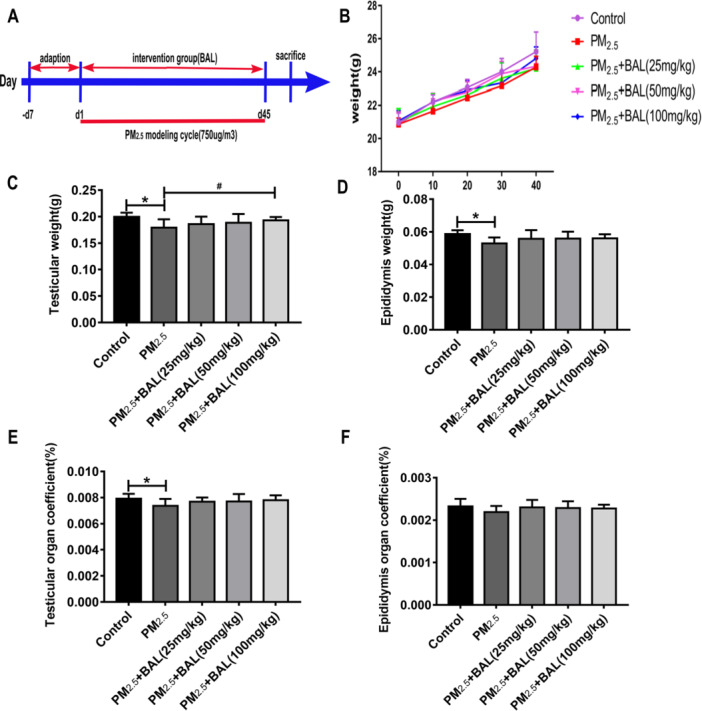
Effects of PM_2.5_ exposure on body weight and reproductive organs of mice. (A) Schematic diagram of PM_2.5_ exposure period. (B) Weekly weight change of mice. (C) Mouse testis weight. (D) Mouse epididymis weight. (E) Mouse testis organ coefficient. (F) Mouse epididymis organ coefficient. The data are expressed as mean ± SD (*n* = 6 per group). *p < 0.05, compared with the control group; ^**#**^ *p* < 0.05, compared with PM_2.5_ exposure group.

A single concentration oral and nasal inhalation exposure system from Beijing Huironghe Technology Co. Ltd was used. The density of PM_2.5_ was measured by the filter membrane weighing method. Mice in the experimental group were exposed to 750 ug/m^3^ at a fixed time for 4 h every day [26], while the control group mice were exposed to the same experimental conditions without PM_2.5_ exposure for 45 days (Figure 1A). The weight of mice determined the volume of gavage, and baicalin gavage was conducted 1 h before PM_2.5_ exposure [20]. It was mixed well when used and stored in the refrigerator at −20°C.

### 2.5 Body Weight and Organ Coefficient Analysis

After inducing cervical spondylolysis, the mice were killed, and their testes were dissected and separated immediately to remove the excess fat and mucosa on the surface. The organ coefficients of the testes and epididymis were weighted and calculated (organ coefficient = organ weight/body weight × 100%).

### 2.6 Sperm Count

The epididymis was put in a test tube containing 5 mL of normal saline, The epididymis was cut into pieces and put in a 37°C water bath for 10 min to keep sperm vitality. 10 μL sperm suspension was dropped into the blood cell counting plate. After the square was found under the low‐power microscope, the upper left, lower left, upper right, and lower right in the counting room were counted using the high‐power microscope. Finally, the number of sperm in each milliliter of semen was counted.

### 2.7 HE Staining and Electron Microscopy Analysis

The testis tissue was extracted, fixed with 4% paraformaldehyde for 72 h immediately, and embedded in paraffin. The slices (4 μm) with hematoxylin and eosin (HE) staining were collected, and one side of the testes of two mice in each group was randomly selected for observation under the light microscope. The other side of the testicle was taken and put into the electron microscope fixative at 4°C for 2–4 h, then dehydrated with ethanol, embedded, sliced (60–80 nm), and finally observed under the transmission electron microscope. The images were collected for analysis.

### 2.8 Enzyme Linked Immunosorbent Assay

An ELISA kit (Wuhan, China) was used to detect testosterone levels while ensuring blood collection was carried out at the concentration time and avoiding hemolysis. The absorbance (OD) value was measured at 450 nm for numerical analysis.

### 2.9 Real‐Time Quantitative Polymerase Chain Reaction (RT‐PCR) Analysis

Trizol was used to extract the total RNA of Mouse testicular tissue (*n* = 6 per group). After adding Trizol, the PrimeScript RT kit was used to synthesize cDNA. The cDNA template and primer were amplified with SYBR Premium Ex Taq II and the ABI 7500 real‐time PCR instrument. GAPDH was used as an internal reference gene for quantitative analysis. The mRNA names and primer sequences are shown in Table 1.

**Table 1:** The mRNA names and primer sequences.

Gene	Primer sequence(5′−3′)	Length (bp)
*WNT4*	*Forward ATTGACGGCTGCGAGCTACT*	104
	*Reverse AAGCAGCACCAGTGGAACCT*	
*DKK1*	*Forward AGCCAGTGCCACCTTGAACT*	130
	*Reverse CTGGTACTTGTTCCCGCCCT*	
*LRP6*	*Forward GAGCGAGGGTACATGTACTTTA*	163
	*Reverse CACAGCTTTCGATTCGCTTTAG*	
*β‐catenin*	*Forward ATGCAGATCCCATCCACGCA*	184
	*Reverse TTGCACGTGTGGCAAGTTCC*	
*STAR*	*Forward GGCATACTCAACAACCAGGAAGGC*	330
	*Reverse CTCCATGCGGTCCACAAGTTCTTC*	
*CYP11A1*	*Forward GCCAGCATCAAGGAGACACTGAG*	105
	*Reverse ACGAAGCACCAGGTCATTCACAG*	
*CYP17A1*	*Forward CAAGCCAAGATGAATGCAGA*	129
	*Reverse AGGAAAGCCAGGATCCAGTT*	
*GAPDH*	*Forward GTTGTCTCCTGCGACTTCA*	122

### 2.10 Western Blot

Place the testicular tissue(mass 10 mg) into a pre‐prepared centrifuge tube containing two steel beads. Subsequently, pre‐chilled RIPA buffer supplemented with 1% phosphatase inhibitor is added to the tube. The tissue is then homogenized using a tissue homogenizer. Following homogenization, the resulting lysate is centrifuged at 12,000 × g and 4°C for 15 min, after which the supernatant containing total protein is collected. Protein concentration is quantified using a BCA Protein Assay Kit (Beyotime, Shanghai, China). Equal amounts of protein (50 μg per sample) are subsequently separated via SDS‐PAGE electrophoresis, and then transferred onto a 0.22 μm nitrocellulose (NC) membrane. After being blocked with 5% nonfat milk in Tris‐Buffered Saline and Tween 20 (TBST), the proteins were incubated with Wnt4(1:500, Abcam, USA), LRP6 (1:1000, Proteintech, China), β‐catenin (1:2000, Proteintech, China), StAR (1:500, ABclonal, China), CYP11A1 (1:1000, ABclonal, China) and CYP17A1 (1:1000, ABclonal, China), β‐actin (1:20000, ABclonal, China) antibodies overnight at 4°C, and then with the secondary antibody at room temperature for 1 h. The proteins were detected using a Tanon High‐sig ECL kit following the product instructions. Gray values were analyzed by Image‐J, and the fold expression was taken as the relative protein level, using β‐actin as the internal reference.

### 2.11 Statistical Analysis

All quantitative results are presented as mean ± standard deviation (SD). Statistical analysis was conducted using SPSS 22.0 software, and charts were constructed using GraphPad Prism8.0 software. Multiple group comparisons were performed using one‐way ANOVA, followed by LSD post‐hoc test after confirming normal distribution and equal variance. Statistical significance was set at *p* < 0.05.

## 3 Results

### 3.1 Effects of Baicalin on Body Weight and Reproductive Organs of Mice Exposed on PM_2.5_

The influence of PM_2.5_ exposure on the body weight of male mice is shown in Figure 1B. The weight of mice in each group was relatively uniform before exposure, and there was no significant difference in body weight in each group each week after exposure for 45 days (*p* > 0.05).

Our model demonstrates that PM_2.5_ exposure is harmful to male reproductive organs (Figure 1C,D). Testis and epididymis weight decreased in the PM_2.5_ exposure group compared with the control group (*p* < 0.05). In the baicalin group, testis weight increased in the 100 mg/kg group (*p* < 0.05), but there was no significant difference in the epididymis group (*p* > 0.05). Compared with the control group, the testis organ coefficient decreased in the PM_2.5_ exposure group (*p* < 0.05). After baicalin treatment, testis and epididymal organ coefficients did not change(*p* > 0.05; Figure 1E,F). These results indicate that baicalin may have a certain easing effect on the changes intesticular weight and organ coefficient induced by PM_2.5_ exposure in mice.

### 3.2 Effects of Baicalin on Sperm Count in Mice Exposed to PM_2.5_

**Figure 2:**
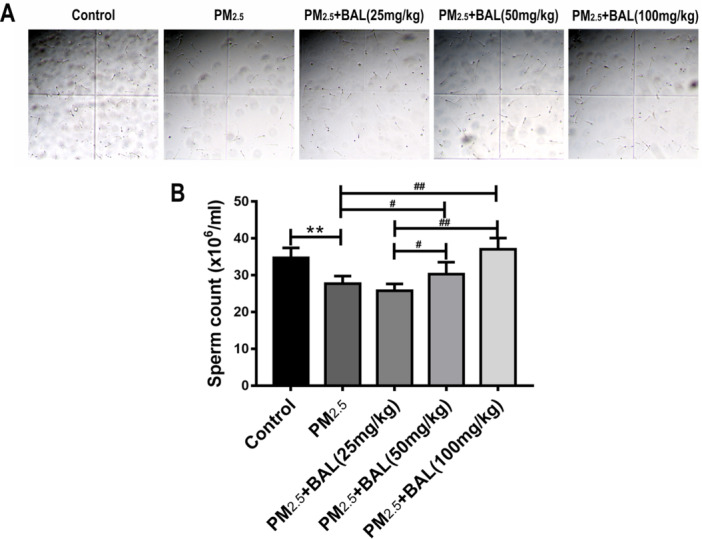
Effect of baicalin on the sperm number of mice exposed to PM_2.5_. (A) Micrograph of mouse sperm. (B) Changes of sperm number in mice. The data are expressed as mean ± SD (*n* = 6 per group). **p < 0.001, compared with the control group;^**#**^ *p* < 0.05 and ^**##**^ *p* < 0.001, compared with PM_2.5_ exposure group.

Studies have shown that PM_2.5_ can damage male germ cells and adversely affect sperm synthesis, quantity, vitality, etc [27]. Our results showed that mice exposed to PM_2.5_ had significantly reduced sperm counts, increased dead cells, and tailless (*p* < 0.05). Baicalin treatment resulted in increased sperm counts and distribution of tailed spermatozoa, and significantly improved viability (*p* < 0.05; Figure 2A,B).

### 3.3 Effects of Baicalin on Pathological and Electron Microscopic Changes of Testis in Mice Exposed to PM_2.5_

**Figure 3:**
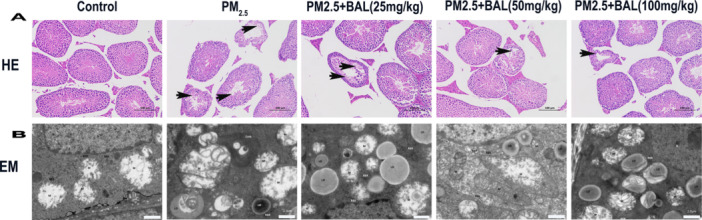
Effect of baicalin on pathological and electron microscopic changes of testes in mice exposed to PM_2.5_. (A) Histopathological analysis of mouse testis (200x) Scale:100 µm. Arrow represents atrophy of seminiferous tubules. (B) Transmission electron microscope analysis of mouse testis (x5.0k) Scale: 2.0 µm. M: Mitochondria, RER: endoplasmic reticulum, N:nucleus, LD:lipid droplet, ASS: autophagic lysosome, Cmb: membranous body.

According to our pathological findings (Figure 3A), the testis parenchyma of the control group was composed of multiple curved and slender spermatogenic tubules, with 4–5 layers of spermatogenic epithelial cells in regular arrangement and abundant spermatozoa in the lumen. In the PM_2.5_ exposure group, multiple spermatogenic tubule atrophy was observed, spermatogenic epithelial cells were reduced, and the number of spermatogenic sperm was significantly reduced. After baicalin treatment, one spermatogenic tubule atrophy was observed occasionally, and the number of spermatogenic sperm in the surface and cavity gradually increased. Further electron microscopic analysis showed that, compared to the control group, mitochondria in the PM_2.5_ exposure group were severely swollen, rough endoplasmic reticulum (RER) was fragmented and dispersed, and lipid droplets (LD) were locally aggregated with an uneven electron density was, partly accompanied by myeloid structures. After baicalin treatment, there were few collagen fibers around, relatively few mitochondria (M), a few mild swellings, orderly cristae, uniform electron density, and occasional autophagic lysosomes (ASS) in the cells (Figure 3B).

### 3.4 Effect of Baicalin on Testosterone Level and Gene Expression of Testosterone Synthesis Pathway in Mice Exposed to PM_2.5_

**Figure 4:**
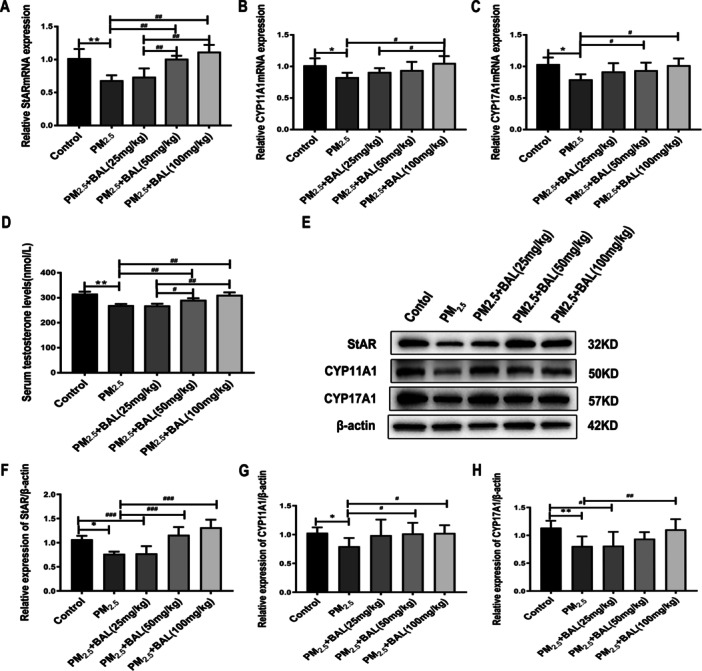
Effect of baicalin on testosterone level and gene expression of testosterone synthesis pathway in mice exposed to PM_2.5_. (A–C) Baicalin increased the expression of StAR, CYP11A1, and CYP17A1 mRNA in testis of mice exposed to PM_2.5_. (D) Effect of baicalin on testosterone content in serum of mice exposed to PM_2.5_. (E) StAR, CYP11A1, CYP17A1, and β‐actin representative protein band. (F–H) Effect of baicalin on StAR, CYP11A1, and CYP17A1 protein expression. The data are expressed as mean ± SD (*n* = 6 per group). *p < 0.05 and **p < 0.001, compared with the control group; ^**#**^ *p* < 0.05 and ^**##**^ *p* < 0.001, compared with PM_2.5_ exposure group.

The testosterone level in the PM_2.5_ exposure group was 267.61 ± 7.54 nmol/L. Compared with the control group 313.14 ± 11.30 nmol/L, The testosterone level was significantly declined (*p* < 0.05), After the intervention of baicalin, the testosterone levels were significantly improved. In the 100 mg/kg baicalin group was restored to 308.77 ± 22.32 nmol/L (*p* < 0.05; Figure 4D). The testosterone synthesis genes StAR, CYP11A1, and CYP17A1 were significantly down‐regulated (*p* < 0.05) in the PM_2.5_ exposure group. Compared to the PM_2.5_ exposure group, the genes and proteins related to testosterone synthesis, including StAR, CYP11A1, and CYP17A1, were significantly increased after baicalin intervention, especially in the 100 mg/kg group (Figure 4). These results indicate that baicalin can restore testosterone levels and expression of testosterone synthesis‐related proteins in mice.

### 3.5 Effects of Baicalin on DKK1‐Wnt/β‐catenin Signaling Pathway in Testicular Tissue of Mice Exposed to PM_2.5_

**Figure 5:**
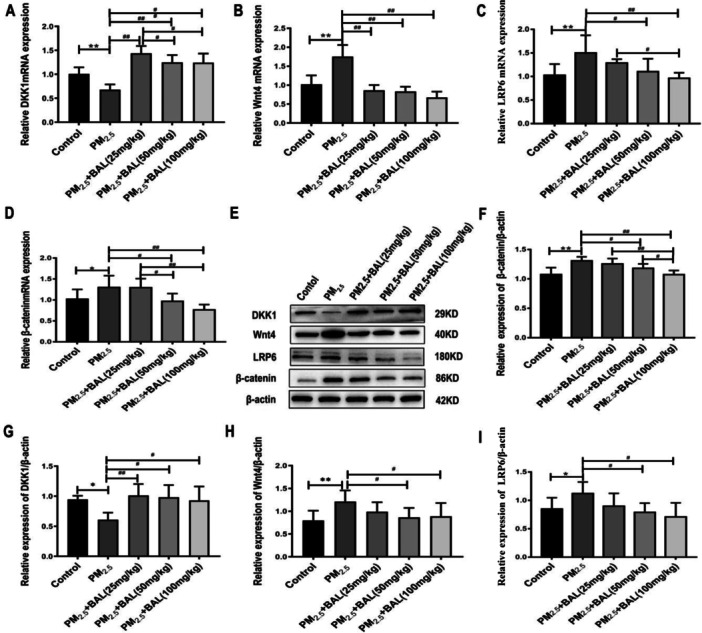
Effects of baicalin on Wnt/β‐catenin signaling pathway in testicular tissue of mice exposed to PM_2.5_. (A–D) Effect of baicalin on DKK1, Wnt4, LRP6, and β‐catenin mRNA expression. (E) DKK1, Wnt4, LRP6, β‐catenin, and β‐actin representative protein band of actin. (F–I) Effect of baicalin on DKK1, Wnt4, LRP6, and β‐catenin protein expression. The data are expressed as mean ± SD (*n* = 6 per group). *p < 0.05 and **p < 0.001, compared with the control group; ^**#**^ *p* < 0.05 and ^**##**^ *p* < 0.001, compared with PM_2.5_ exposure group.

In order to investigate the effect of baicalin, we first detected the expression of DKK1 in testicular tissue. Compared with the control group, the DKK1 mRNA in the PM_2.5_ exposure group was significantly down‐regulated. After baicalin treatment, it was significantly up‐regulated (*p* < 0.05; Figure 5A), Meanwhile, the protein expression of DKK1 in baicalin groups was significantly higher than that in PM_2.5_ exposure group (*p* < 0.05), and there was no significant difference among baicalin groups (Figure 5E,G). In addition, the role of Wnt/β‐catenin pathway in the testis was examined. Compared with the PM_2.5_ exposure group, Wnt4, LRP5/6, and β‐catenin mRNA were significantly down‐regulated in baicalin groups, especially in the 50 and 100 mg/kg baicalin groups (*p* < 0.05; Figure 5B–D). Furthermore the protein expressions of theWnt4, LRP5/6, and β‐catenin were significantlyincreased in the PM_2.5_ exposure group (*p* < 0.05), while these proteins were significantly decreasedin the 50 mg/kg and 100 mg/kg baicalin groups (*p* < 0.05) (Figure 5E,F,H,I). These results indicate that baicalin may be able to regulate the DKK1‐Wnt/β‐catenin signaling pathway to protect male reproduction from PM_2.5_ exposure.

## 4 Discussion

PM_2.5_ is a ubiquitous environmental pollutant. According to the research on PM_2.5_ pollution mortality assessment, about 32% of the total deaths in China in 2013 were related to PM_2.5_ [28]. PM_2.5_ as one of the main components of the atmosphere, is related to male reproductive diseases [29]. Baicalin is a major flavonoid compound extracted from the dried root of Scutellariabaicalensis Georgi [30]. It has no obvious toxic or side effects [31]. Our study exposed mice to a concentration of 750 ug/m^3^, which is 10 times the limit value of PM_2.5_ in China. Intragastric administration of baicalin to mice 1 h before PM_2.5_ exposure. We found that baicalin has a certain improvement effect on the reproductive system damage induced by PM_2.5_ exposure in mice, and this improvement effect may be related to the DKK1‐Wnt/β‐catenin signaling pathway.

Previous studies have shown that the quality of male sperm has declined year after year, and the incidence of infertility has also increased [32, 33, 34]. In a study of men in France, semen concentrations continued to fall, as did the proportion of normal‐form sperm [35]. Our research revealed that PM_2.5_ exposure can significantly reduce the weight of testis and epididymis and the organ coefficient of testis in mice, and decreased testosterone levels. Furthermore, we found that the number of sperm in the PM_2.5_ exposed group was significantly reduced. The pathological and electron microscopic analysis demonstrated that the number of sperm in the cavity decreased, the seminiferous tubules atrophied, and the number of seminiferous epithelial cells decreased, which were consistent with previous research [36]. These findings may be related to the fact that PM_2.5_ exposure causes DNA damage in testicular tissue [37], decreased testosterone levels, significant reduction in sperm count and density, and decreased sperm concentration and motility in mice [36, 38]. In addition, we also found that the damage changes mentioned above were alleviated after baicalin intervention. The weight of the testis increased, the number of sperm gradually increased on the surface and in the cavity, a few mitochondria were slightly swollen, and the ridge was arranged neatly. This might be related to the antibacterial and anti‐inflammatory effects of baicalin [39, 40].

Studies have shown that the decrease of sperm concentration and motility caused by PM_2.5_ exposure may be related to the decrease of testosterone level. The mRNA and protein expression of StAR, CYP11A1, CYP17A1 mRNA which are closely related to testosterone synthesis, are significantly decreased after high concentration of PM_2.5_ exposure [10, 11, 36]. Our findings are consistent with those of previous studies. Furthermore, after baicalin intervention, these genes and proteins were significantly increased to alleviate the damage caused by PM_2.5_. This protect effect may be related to the fact that baicalin can inhibit the down‐regulation of StAR, CYP11A1, and CYP17A1 mRNA genes and proteins, thereby significantly increasing testosterone levels, restoring sperm count, and protecting testis from damage.

Studies have demonstrated that the Wnt signaling pathway serves as an essential core regulatory axis for maintaining normal testicular physiological functions [41, 42, 43]. Physiological levels of Wnt pathway activity are the prerequisite for the orderly progression of spermatogenesis. However, aberrant excessive activation of this pathway directly triggers spermatocyte arrest at meiotic prophase I, downregulation of blood‐testis barrier tight junction proteins, disruption of barrier structural integrity and dysregulation of the spermatogenic epithelial microenvironment homeostasis [41, 44, 45]. These pathological cascades ultimately induce spermatogenic defects and impair male fertility. Wnt4 is a specific ligand of the Wnt/β‐catenin signaling pathway, while LRP5 and LRP6 are the the canonical cell surface co‐receptors of the Wnt pathway [41, 46]. Upon the binding of Wnt4 to LRP5/6, Axin is recruited to the plasma membrane and subsequently targeted for ubiquitination‐mediated degradation. This process functionally disrupts the cytoplasmic β‐catenin destruction complex, leading to the activation of canonical Wnt signaling [14, 16]. As an essential scaffold protein, Axin orchestrates the ordered phosphorylation and ubiquitination‐dependent degradation of β‐catenin by forming a multi‐protein complex with APC, β‐catenin, GSK3β and CK1. Ultimately, the abnormally accumulated β‐catenin translocates into the nucleus, where it interacts with the transcription factor T‐cell factor/lymphoid enhancer factor (TCF/LEF) to initiate the transcription of downstream target genes [16, 41]. DKK1 is a highly conserved secreted glycoprotein that functions as a potent endogenous negative regulator of the canonical Wnt pathway [47]. Accumulating evidence demonstrated that DKK1 exerts robust inhibitory effects on the canonical Wnt/β‐catenin cascade by competitively blocking the interaction between Wnt ligands and their membrane co‐receptors LRP5/6 [48]. Dysregulation of DKK1 has been tightly linked to a broad spectrum of human diseases, including osteoporosis [49], rheumatoid arthritis [50], Alzheimer's disease [51], and complications of diabetes [52]. However, there are no reports yet on whether this expression is related to the damage to the reproductive system of mice exposed to PM_2.5_. The results of this study showed that mice exposed to PM_2.5_ for a specified period, the gene and protein expression levels of DKK1 in their testicular tissues were significantly decreased, while the expression levels of Wnt4, LRP6, and the downstream effector molecule β‐catenin were significantly increased. This result suggests that during the process of testicular tissue damage induced by PM_2.5_ exposure, the downregulation of DKK1 may be a key triggering factor for the abnormal excessive activation of the Wnt/β‐catenin pathway. After baicalin intervention, the expression level of DKK1 in testicular tissue was significantly upregulated. It is speculated that baicalin may exert a male reproductive protective effect by upregulating DKK1 expression, restoring the inhibitory regulation of the Wnt/β‐catenin pathway, and thus alleviating the pathological damage of testicular tissue induced by PM_2.5_. However, Consistent with the designs of several studies in the same field [53, 54], we did not detect the downstream transcriptional activity of the Wnt/β‐catenin pathway (e.g., TCF/LEF reporter activity or target genes). This limitation partially weakens the completeness of the mechanistic evidence chain. Therefore, in future research on this topic, it is necessary to strengthen the detection of TCF/LEF reporter gene activity.

## 5 Conclusion

The results showed that baicalin could improve the severe mitochondrial swelling, RER fragmentation and dispersion, and lipid droplet aggregation observed in the testes of mice after PM_2.5_ exposure. It significantly reduced the pathological damage caused by PM_2.5_ exposure to the testes of mice. After intervention with baicalin, the expression of testosterone synthesis genes and proteins StAR, CYP11A1, and CYP17A1 in mice can be increased, promoting testosterone synthesis, improving the atrophy of testicular spermatogenic tubules, increasing the number of sperm in mice, and enhancing sperm motility. In addition, our research also revealed that these improvement effects of baicalin on the reproductive system may be associated with the DKK1‐Wnt/β‐catenin signaling pathway. These findings explore the role and potential mechanism of baicalin in improving the reproductive system damage caused by PM_2.5_ exposure in mice. However, despite the promising role of baicalin as a safe and natural flavonoid in traditional Chinese medicine, its mechanism of action still needs to be further investigated.

## Author Contributions

**Lili Deng:** project administration, methodology, supervision, writing – original draft, writing – review and editing. **Juan Wang:** writing – original draft, data curation, visualization, formal analysis, project administration. **Shuyin Li:** conceptualization, methodology. **Yaping Mao:** writing – review and editing, methodology. **Ye Sun:** conceptualization, methodology. **Qiaoqiao Yang:** formal analysis, project administration. **Zhenghui Xie:** project administration, formal analysis. **Chen Zhang:** project administration. **Shuo Gong:** project administration. **Chunling Xiao:** methodology, conceptualization, supervision, project administration, resources, writing – review and editing. **Mingyue Ma:** conceptualization, project administration, resources, methodology, writing – review and editing, supervision.

## Conflicts of Interest

The authors declare no conflicts of interest.

## Data Availability

Journal of Applied Toxicology (JAT) The datasets generated and analyzed during the current study are available from the corresponding author upon reasonable request.
